# Depression Prevalence, Screening, and Treatment Rates in Adolescents with Obesity in Ambulatory Settings

**DOI:** 10.1192/j.eurpsy.2025.675

**Published:** 2025-08-26

**Authors:** L. Li

**Affiliations:** Psychiatry, University of Alabama at Birmingham, Birmingham, United States

## Abstract

**Introduction:**

Obesity is a growing problem in several developed countries and has a complex etiology in teenagers. Approximately one-third of children and adolescents in the United States are overweight or obese. However, it is not clear how depression and obesity are screened and treated in the primary care setting for adolescents.

**Objectives:**

This study aims to describe the prevalence, screening, and treatment rates for depression in adolescents in ambulatory settings in the United States.

**Methods:**

Data on 444,080,295 male and female adolescents ages 13-18 were extracted from the 2008-2018 CDC National Ambulatory Medical Care Survey datasets. Adolescents were stratified by weight groups based on CDC guidelines (i.e., body mass index percentile).

**Results:**

Of the adolescents, 16.89% were obese, 13.81% were overweight, 43.39% were normal weight, and 25.91% were underweight. Depression screening rates in adolescents with obesity is 2.89%, overweight is 3.35%, normal weight 3.49%, and underweight is 2.83% (*p*=0.382). Prevalence of depression in adolescents with obesity is 7.17%, overweight is 6.04%, normal weight is 6.31%, and underweight is 12.14% (*p*<0.0001). Prevalence of counseling and psychotherapy in adolescents with obese status is 2.70%, overweight status is 2.89%, normal weight is 2.92%, and underweight is 11.27% (*p*<0.0001). Patients seen by primary care health workers, age, female gender, number of chronic conditions, and increased visits are significant predictors of depression diagnosis in adolescents.

**Conclusions:**

Depression in adolescents who are overweight or obese is under-screened for, under-identified, and under-treated. More mental health counseling and psychotherapy must be offered to those with both depression and obesity.

**Disclosure of Interest:**

None Declared

